# Electrochemical Oxidation of EDTA in Nuclear Wastewater Using Platinum Supported on Activated Carbon Fibers

**DOI:** 10.3390/ijerph14070819

**Published:** 2017-07-21

**Authors:** Bo Zhao, Wenkun Zhu, Tao Mu, Zuowen Hu, Tao Duan

**Affiliations:** 1 Key Subject Laboratory of National Defense for Radioactive Waste and Environmental Security, Southwest University of Science and Technology, Mianyang 621010, China; bozhao@swust.edu.cn (B.Z.); duantao112@163.com (T.D.); 2 School of Life Science and Engineering, Southwest University of Science and Technology, Mianyang 621010, China; 3 Sichuan Civil-Military Integration Institute, Mianyang 621010, China; 4 China Academy of Engineering Physics, Mianyang 621000, China; mutao1224@126.com (T.M.); huzuowen1979@sina.com (Z.H.)

**Keywords:** ethylenediaminetetra-acetic acid, electrochemical oxidation, Pt/ACF electrode, degradation intermediates

## Abstract

A novel Pt/ACF (Pt supported on activated carbon fibers) electrode was successfully prepared with impregnation and electrodeposition method. Characterization of the electrodes indicated that the Pt/ACF electrode had a larger effective area and more active sites. Electrochemical degradation of ethylenediaminetetra-acetic acid (EDTA) in aqueous solution with Pt/ACF electrodes was investigated. The results showed that the 3% Pt/ACF electrode had a better effect on EDTA removal. The operational parameters influencing the electrochemical degradation of EDTA with 3% Pt/ACF electrode were optimized and the optimal removal of EDTA and chemical oxygen demand (COD) were 94% and 60% after 100 min on condition of the electrolyte concentration, initial concentration of EDTA, current density and initial value of pH were 0.1 mol/L, 300 mg/L, 40 mA/cm^2^ and 5.0, respectively. The degradation intermediates of EDTA in electrochemical oxidation with 3% Pt/ACF electrode were identified by gas chromatography-mass spectrum (GC-MS).

## 1. Introduction

The main purpose for considering the use of the decontamination techniques in the nuclear installation out of commission is removing the contamination from equipment and to reduce dose levels. Ethylenediaminetetra-acetic acid (EDTA) is an efficient chelating agent widely used in industrial, agricultural and pharmaceutical applications, among others. It is also an important decontaminating agent in the nuclear industry. The presence of EDTA in radioactive liquid waste made it difficult to treat because: (1) EDTA of ionic state will form to complex with radioactive nuclide and this made it difficult to be separated. (2) The existence of EDTA in the radioactive wastewater reduced chemisorption of radioactive nuclide and accelerated its migration rate in ground water, soil and surface water [1]. (3) The degradation of EDTA in radioactive wastewater will generate CO, CO_2_, H_2_ and these gases decreased stability of wastewater [2]. The conventional methods to treat EDTA in radioactive wastewater such as incineration, pyrolysis, Fenton oxidation, wet oxidation [3], ultraviolet light and ozone oxidation [4], are low efficiency and require complicated devices. Nowadays, electrochemical oxidation systems have been proven to be very effective to treat a variety of organic wastewaters [5,6,7]. 

Electrochemical advanced oxidation processes are environmentally friendly technologies capable of producing ·OH (hydroxyl radicals). In the electrochemical oxidation process, the solutions are decontaminated through the direct reaction of pollutants with ·OH radicals formed at the anode surface during the electrolysis process [8]. As we all know, the anode material plays an important role in the electrochemical processes for the pollutant removal efficiency [9,10,11]. Many electrode materials, such as Ti/RuO_2_, nano-ZnO, IrO_2_, diamond and tungsten trioxide-exfoliated graphite composite [12,13,14,15,16], have been employed to the electrochemical degradation of different organic pollutants. Despite the high efficiency for organic compounds degradation, however, such electrochemical processes are usually energy-intensive because of high overpotential and side reactions [17]. This has attracted more researchers in searching for more efficient and specific catalysts. Recently, metal nanoparticles are recognized as high-efficiency catalysts and have been used for organic pollutant degradation. For example, nano-scale Cu, Au, Ag and Fe-Ni, are known to efficiently catalyze redox reactions with a high stability [18,19,20,21].

In this study, a novel Pt/ACF electrode (platinum supported on activated carbon fibers) was prepared by two methods and was used to degrade EDTA. Compared with Pt sheet electrode, the Pt/ACF electrode showed an obviously better effect on EDTA and chemical oxygen demand (COD) removal. The structure and morphology of the electrode was studied by scanning electron microscopy (SEM) and nitrogen adsorption. The experimental parameters, including the electrolyte concentration, initial EDTA concentration, current density and initial value of pH, were studied and optimized. The oxidation intermediates were identified by gas chromatography-mass spectrum (GC-MS).

## 2. Experimental

### 2.1. Materials and Reagents

Na_2_SO_4_, Na_2_EDTA·2H_2_O, H_2_SO_4_, NaOH and H_2_PtCl_6_·6H_2_O, which are commercially available, were used without further purification. Activated carbon fibers (ACF) were obtained from Nantong Senyou Carbon Fiber Company Limited (Nantong, China). Platinum sheet (Pt) electrode was obtained from Tianjin Aida Technology Development Company (Tianjin, China).

### 2.2. Electrode Preparation and Characterization

The detailed procedure for the preparation of the Pt/ACF electrode has been presented in previous works [22]. Briefly, ACF was first washed in deionized water, and then boiled in concentrated HCl solution for three days to remove impurity. Lastly, the ACF was thoroughly rinsed with deionized water to remove residual HCl, and then dried in the oven at 100 °C. Two methods were used for the catalyst preparation: incipient wetness impregnation and electrodeposition. For the conventional impregnation method, an aqueous solution of H_2_PtCl_6_ was used as precursor and two platinum loadings (3 wt % and 5 wt %) were prepared. A piece of ACF (1 cm × 1.5 cm) was first soaked in platinum precursor solution to saturate the ACF pores. The wet ACF was dried under room conditions, then vacuum-dried at room temperature, and finally reduced in a fixed-bed flow reactor with H_2_ (30 mL/min) at 400 °C for 2 h to give Pt/ACF catalyst. For electrodeposition method, 3 wt % platinum loadings were prepared (EDPt/ACF), a piece of ACF (0.1 g) was fixed with electrode holder as cathode, films of metal catalysts were formed on the ACF surface by thoroughly galvanostatic deposition using aqueous H_2_PtCl_6_ solutions of given concentration (0.008 g H_2_PtCl_6_·6H_2_O in 10 mL water, Pt content 0.003 g) with a current of 1 A. Pt sheet was used as the counter electrode. ACF electrode was placed in the middle of the cell and electrolyte was stirred with magnetic stirrer during electrodeposition.

The prepared electrodes were comprehensively characterized. Specifically, the surface morphology of the ACF, 3% Pt/ACF, 5% Pt/ACF and 3% EDPt/ACF electrodes were characterized using scanning electron microscopy (Sirion200, FEI Ltd., Eindhoven, The Netherlands). Their pore structure was evaluated with the adsorption/desorption isotherms of N_2_ using an automatic surface area and pore analyzer (Tristar II 3020 M, Micromeritics Co., Atlanta, GA, USA). The dispersion of Pt was checked by CO adsorption [23].

### 2.3. Electrochemical Experiments

The Pt/ACF electrode was used as the anode and a titanium plate was used as the cathode. EDTA was selected as the model organic pollutant. Firstly, different electrodes were used to study the effect of electrode type on the EDTA and COD removal. Then, the influencing parameters, including electrolyte concentration (0.05–0.5 mol/L), initial EDTA concentration (100–1000 mg/L), the initial pH value (3.0–11.0) and current density (10–50 mA/cm^2^), were studied with 3% Pt/ACF electrode.

### 2.4. Analytical Methods

Quantitative analysis of EDTA was measured by HPLC method according to the procedure reported by Li et al. [24]. The instantaneous current efficiency (ICE) was calculated on the basis of chemical oxygen demand of the reaction solution, using the following equation [25]:

(1) $$\mathrm{ICE}=\frac{\left({\mathrm{COD}}_{0}-{\mathrm{COD}}_{t}\right)}{8It}FV\times 100\%$$

In this equation, COD_0_ and COD_t_ (g/L) are COD at time intervals. *F* is the Faraday constant (96,487 C/mol), *V* is the volume of the solution (L), *I* is the current (A) and *t* is the reaction time (s).

The degradation products of EDTA were analysed by GC-MS according to the procedure reported by Li et al. [26].

## 3. Results

### 3.1. Characterization of Pt/ACF Electrode

**Figure 1:**
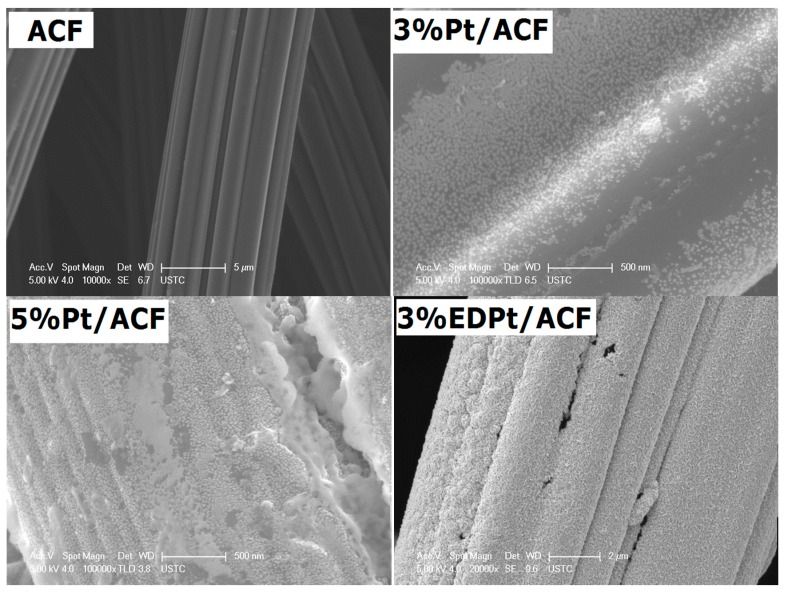
SEM images of the ACF, 3% Pt/ACF, 5% Pt/ACF and 3% EDPt/ACF electrodes. SEM: scanning electron microscopy; ACF: activated carbon fibers.

The surface morphology of the prepared Pt/ACF electrodes were characterized. Figure 1 shows the SEM images of the black ACF, 3% Pt/ACF, 5% Pt/ACF and 3% EDPt/ACF. As we have reported before [22], the conductivity of ACF is very high because of its network structure. The specific surface area (BET) of blank ACF is 2398 m^2^/g, very similar to the value that has been reported [27]. Compared with other supporting materials [28], ACF has much larger surface area. As Table 1 shows, the majority pores of ACF are micropores with the volume being 1.13 cm^3^/g. Meanwhile, the micropore volumes of ACF decreased with loading different content of platinum; this decrease might be attributed to the fact that the pores on the ACF were partially filled with platinum. Among all the electrodes, the surface area and micropore volume of 3% EDPt/ACF are minimum, this is probably due to the fact that pores on the ACF surface were filled with platinum, leading to a metal film (Figure 1). As seen in Figure 1, most of the platinum was uniformly distributed as nano-lattice in 3% Pt/ACF, but partial platinum was agglomerated to bulk in 5% Pt/ACF. As shown in Table 2, when metal loading increased from 3 to 5%, dispersion and Pt area all decreased sharply. However, the particle size increased from 2.1 to 6.5 nm. 

### 3.2. Effect of Different Electrodes

**Figure 2:**
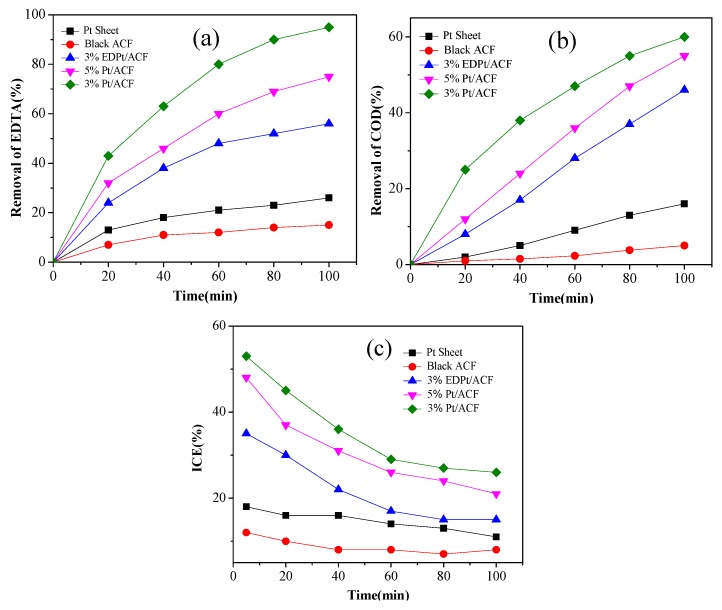
The effect of different electrodes on EDTA removal (**a**) concentration; (**b**) COD; (**c**) ICE (initial pH 5; initial EDTA concentration 300 mg/L; current density 40 mA/cm^2^; electrolyte concentration 0.1 mol/L). EDTA: ethylenediaminetetra-acetic acid.

To explore the electrochemical oxidation performance of different Pt/ACF electrodes, black ACF, Pt sheet, 3% Pt/ACF, 5% Pt/ACF and 3% EDPt/ACF were prepared to degrade EDTA on condition of the current density, electrolyte concentration, EDTA concentration were 40 mA/cm^2^, 0.1 mol/L and 300 mg/L, respectively. As were shown in Figure 2, with the anodes of Pt sheet, black ACF, 3% Pt/ACF, 5% Pt/ACF and 3% EDPt/ACF electrodes, the effects were 26%, 15%, 94%, 75% and 56% for EDTA removal after 100 min. While for COD removal, the effects were 16%, 5%, 60%, 51% and 47% after 100 min, respectively. Compared with the effects of other electrodes, the removal of EDTA and COD of 3% Pt/ACF electrode was the best. Meanwhile, the ICE of 3% Pt/ACF electrode was the highest in the whole process (Figure 2), suggesting that 3% Pt/ACF electrode possessed an excellent performance for the EDTA degradation.

### 3.3. Effect of Electrolyte Concentration

**Figure 3:**
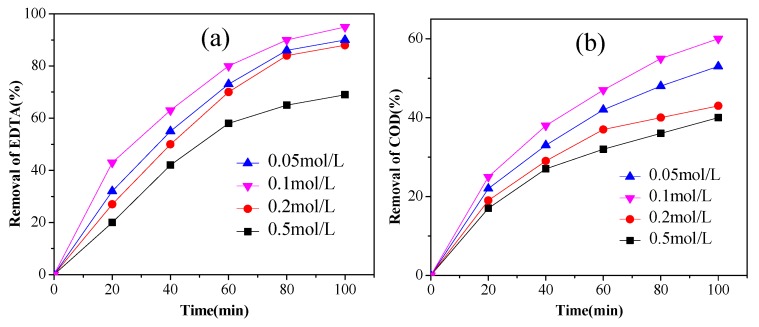
The effect of supporting electrolyte (Na_2_SO_4_) concentration on the EDTA removal of (**a**) concentration, (**b**) COD (3% Pt/ACF electrode; initial pH 5.0; initial EDTA concentration 300 mg/L; current density 40 mA/cm^2^). COD: chemical oxygen demand.

The electrolyte was added to promote the electroconductivity of the EDTA solution [29]. Na_2_SO_4_ was selected as supporting electrolyte varying from 0.05 mol/L to 0.5 mol/L under the condition of the current density 40 mA/cm^2^, pH 5.0 and the EDTA concentration 300 mg/L. As shown in Figure 3, after 100 min electrolysis, the removal of EDTA were 90%, 95%, 88% and 69%, and its COD removal were 53%, 60%, 43% and 40% at Na_2_SO_4_ concentration of 0.05, 0.1, 0.2 and 0.5 mol/L, respectively. As a result, the concentration of Na_2_SO_4_ 0.1 mol/L was chosen as an optimal parameter for the rest of this study.

### 3.4. Effect of Initial Concentration of EDTA

**Figure 4:**
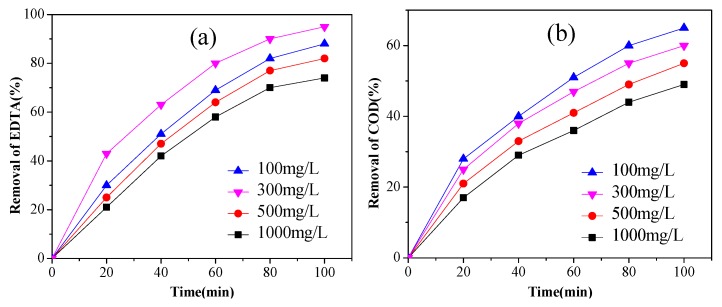
The effect of initial EDTA concentration on EDTA removal (**a**) concentration; (**b**) COD (3% Pt/ACF electrode; current density: 40 mA/cm^2^; electrolyte concentration: 0.1 mol/L).

Maintaining the conditions of pH 5.0, current density 40 mA/cm^2^, electrolyte concentration 0.1 mol/L, the effect of initial concentration of EDTA on degradation was investigated, ranging from 100 to 1000 mg/L. As could be observed in Figure 4, the removal rate of EDTA increased at low concentrations (100 to 300 mg/L) of EDTA but decreased at higher concentrations (500 to 1000 mg/L). The highest value of EDTA removal (95%) was obtained with 300 mg/L initial concentration of EDTA, and the highest value of COD removal (65%) was obtained with 100 mg/L of EDTA. So, the level of 300 mg/L of EDTA was the optimal one.

### 3.5. Effect of pH

**Figure 5:**
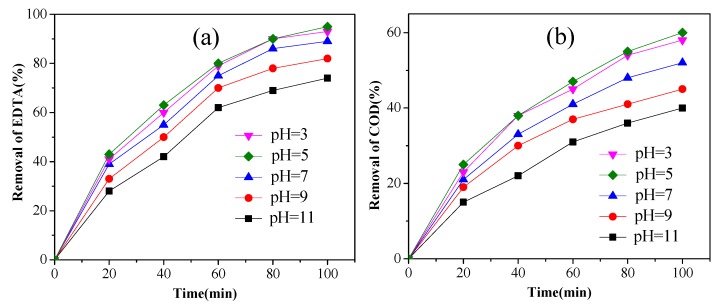
The effect of pH on the on EDTA removal (**a**) concentration; (**b**) COD (3% Pt/ACF electrode; EDTA concentration: 300 mg/L; electrolyte concentration: 0.1 mol/L; current density: 40 mA/cm^2^).

Different pH value, varying from 3 to 11 which contain acid environment and alkaline condition, was selected to study the influence of pH on the removal of EDTA on the condition of current density 40 mA/cm^2^, EDTA concentration 300 mg/L, electrolyte concentration 0.1 mol/L. As was shown in Figure 5, the efficiency of the electrochemical oxidation process increased in acidic condition, a similar outcome was reported by Han and Wang [28]. EDTA removals were both more than 90% at initial pH 3.0 and 5.0 after 100 min of electrolysis. Therefore, pH 5.0 was the optimum pH value.

### 3.6. Effect of Current Density

**Figure 6:**
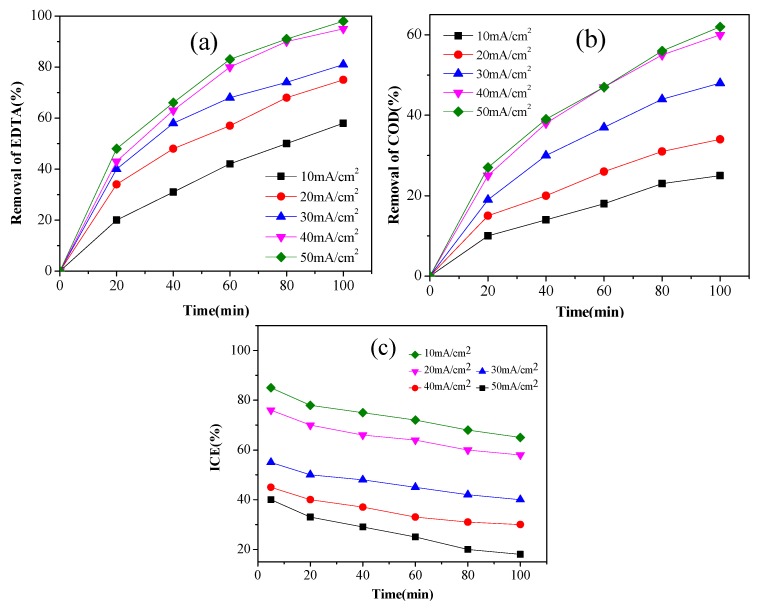
The effect of current density on EDTA removal (**a**) concentration; (**b**) COD; (**c**) ICE (3% Pt/ACF electrode; EDTA concentration: 300 mg/L; electrolyte concentration: 0.1 mol/L; pH 5.0). ICE: instantaneous current efficiency.

To assess the effect of current density on the degradation of 300 mg/L EDTA, current density ranging from 10 to 50 mA/cm^2^ were applied. Figure 6a,b showed that the removal of EDTA and COD all enhanced with the increase of current density from 10 to 50 mA/cm^2^. Figure 6c showed that the ICE decreased with increased current density and it decreased rapidly when current density was over 30 mA/cm^2^. Therefore, taking into account higher efficiency of degradation and better energy utilization ratio, the current density of 40 mA/cm^2^ was chosen in this study.

### 3.7. Identification of the Degradation Products of EDTA

**Figure 7:**
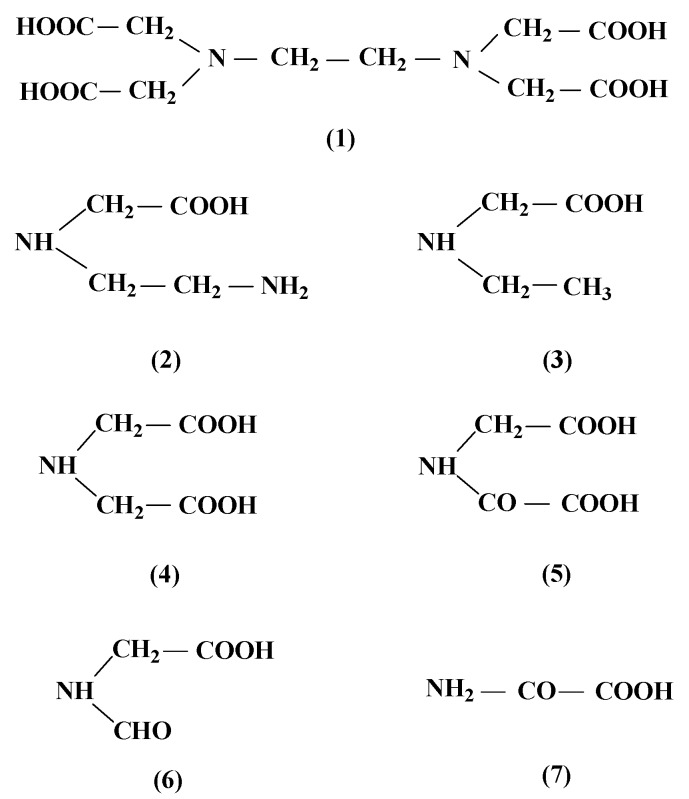
EDTA and electrochemical oxidation intermediates with Pt/ACF electrode.

The intermediate products formed during the degradation process were analyzed by GC-MS technique and the results were shown in Figure 7. In this study, the main oxidation intermediates of EDTA were amino acid.

## 4. Discussion

This work demonstrates that the Pt/ACF can effectively catalyze EDTA degradation. Among various electrodes, the 3% Pt/ACF exhibited the highest EDTA degradation efficiency. 

Dispersion of Pt is summarized in Table 2. It can be seen that both dispersion and metal area decrease sharply with increasing Pt loading from 3 to 5%. However, the particle size shows the opposite trend, these results are consistent with SEM images of the 3% and 5% Pt/ACF. These results indicated that the dispersion of Pt depends strongly on preparation methods and metal loading. In addition, there is a sharp decrease in CO uptake with an increase of Pt loading from 3 to 5%, implying that more active sites are present on 3% Pt/ACF. This is probably the primary reason that 3% Pt/ACF exhibits the best catalytic activity among all electrodes.

The degradation of EDTA in aqueous solution depends on various operational parameters such as concentration of supporting electrolyte, initial EDTA concentration, current density and pH. Na_2_SO_4_ are employed for the degradation of EDTA to allow the flow of electrical current. When the concentration of Na_2_SO_4_ increased from 0.05 mol/L to 0.1 mol/L, the conductivity of the solution enhanced and the removal of EDTA and COD increased [30]. However, when Na_2_SO_4_ concentration increased over 0.1 mol/L, more ${\mathrm{SO}}_{4}^{2-}$ anions were absorbed on the electrode surface, so the number of active site of electrode minimized, causing a decrease tendency in EDTA degradation [31]. Initial concentration of pollutant is another important parameter for the electrochemical oxidation process. In our study, when the initial concentration of EDTA increased, more substrates are transferred to the surface of electrode, the generation of reactive oxidative species (·OH) are limited [32], so excessive EDTA would decrease the degradation efficiency. Many authors have investigated the effect of initial pH on the electrochemical oxidation of pollutants in aqueous medium [30,33]. In this study, low pH was beneficial to degrade the EDTA. This behavior was mainly attributed to the fact that the increase pH of EDTA solution decreased the hydroxylradical generation [34]. Meanwhile, lower pH values diminish the oxygen evolution reaction in favor of organic compound oxidation [35]. Current density is an important parameter in the electrochemical oxidation process. The result of this study indicated that high current density could promote the degradation efficiency, because the generation rate of ·OH was enhanced with the current density rose. However, the side reaction like oxygen evolution would also be intensified at a higher current density. Therefore, choosing a suitable current density is very important in galvanostatic oxidation process.

The GC-MS analysis reveals that the EDTA degradation products were mainly amino acids, some of the compounds have been reported as degradation intermediates of EDTA in the photocatalytic degradation [36] and ozonolysis degradation [37]. Previous studies showed that different intermediates were found in different oxidation processes, some degradation products with functional groups such as carboxylic acids, carbonyl, and amines have been reported [36,38]. Interestingly, the number of intermediates detected in this study is less than that reported previously, possibly because the concentrations of some intermediates are too low to be detected.

## 5. Conclusions

The electrochemical degradation of EDTA over Pt/ACF electrodes was investigated in this study. The major conclusions drawn from this study are summarized as follows:

(1) Two kinds of Pt/ACF electrodes were prepared. The SEM analysis showed that most of the platinum was uniformly distributed as nano-lattice on 3% Pt/ACF. The result of CO adsorption showed that more active sites are present on 3% Pt/ACF; 3% Pt/ACF electrode had a better performance for the removal of EDTA and COD than 5% Pt/ACF and 3% EDPt/ACF electrode.
(2) The experimental parameters which influenced the removal of EDTA and COD were explored using 3% Pt/ACF electrode. The optimal removal of EDTA and COD was 94% and 60% after 100 min electrolysis on condition of electrolyte concentration 0.1 mol/L, initial EDTA concentration 300 mg/L, current density 40 mA/cm^2^ and initial pH value 5.0.
(3) The intermediate generated by electrochemical oxidation of EDTA was detected by GC-MS. It showed that EDTA could be effectively degraded.

## Figures and Tables

**Table 1:** Structural parameters of blank ACF and the catalysts.

Catalyst	Preparation Method	BET (m^2^/g)	Micropore Volume (cm^3^/g)	Micropore Area (m^2^/g)
Blank ACF	Water wash and H_2_ reduction	2398	1.13	1729
3% Pt/ACF	impregnation method	1862	0.94	1347
5% Pt/ACF	impregnation method	1360	0.66	995
3% EDPt/ACF	electrodeposition method	896	0.47	671

BET: specific surface area.

**Table 2:** CO adsorption properties and dispersion of Pt.

Catalyst	CO Adsorption (µmol/g)	Pt Area (m^2^ g/cat)	Pt Area (m^2^ g/Pt)	Dispersion (%)	Particle Size (nm)
3% Pt/ACF	83.4	4.0	134.0	54.2	2.1
5% Pt/ACF	44.8	2.2	43.1	17.5	6.5
3% EDPt/ACF	n.d. ^a^	n.d. ^a^	n.d. ^a^	n.d. ^a^	n.d. ^a^

^a^ No data can be calculated because pores on the ACF surface were filled with Pt.
